# Effect of different cooking conditions on the quality characteristics of chicken claws

**DOI:** 10.1002/fsn3.4197

**Published:** 2024-05-07

**Authors:** Yifan Yu, Xianling Yuan, Zhouyou Zhang, Yidan Zheng, Ying He, Yingru Zhou

**Affiliations:** ^1^ College of Bioengineering Sichuan University of Science and Engineering Zigong China

**Keywords:** chicken claws, cooking conditions, myofibrillar protein, quality

## Abstract

Chicken claw products with their unique texture are loved by consumers, and cooking is a key step to affect the taste of chicken claw consumption, through the moderate hydrolysis of proteins and a series of physicochemical changes, so that the chicken claw gets tender and presents a crispy taste, but the current research on the optimal cooking conditions for chicken claw is still relatively small. In the present work, combinations of time (11, 13, 15, 17, and 19 min) and temperature (82, 86, 90, 94, and 98°C) were applied to the cooking of chicken claws. The effects of different cooking conditions on the quality characteristics of chicken claws were investigated, with special emphasis on the cooking loss rate, color, texture properties, lipid oxidation, myofibrillar fragmentation index (MFI), and total sulfhydryl content. The results showed that the cooking loss rate, lipid oxidation, and MFI value of chicken claws gradually increased, and the total color difference (∆*E*), puncture force, shear force, and total sulfhydryl content gradually decreased with the increase of cooking temperature and cooking time. Overall, chicken claws cooked at 86, 90, and 94°C for 15 and 17 min had better texture and flavor.

## Abstract 2

Cooking treatment had a significant effect on the quality characteristics of chicken claws. Overall, chicken claws cooked at 86, 90 and 94°C for 15 and 17 min had better texture and flavor.

## 1 INTRODUCTION

Chicken claws are a major by‐product of broiler production, but they are processed into different foods and have become a popular food resource in many countries. In addition, the claws are rich in collagen, calcium, cartilage, and chondroitin glucosamine (Gajda et al., 2022), all of which are easily absorbed by the body and can moisturize the skin, promote cell regeneration, and are useful for protecting the skin. Meanwhile, chicken claws are also rich in polyunsaturated fatty acids (Santana Neto et al., 2021), which can enhance immunity and protect internal organs.

Water bath cooking is one of the most commonly used and conveniently used methods in meat processing, which allows moderate hydrolysis of meat nutrients, which is beneficial for human absorption, and which is in line with the concept of healthy diet (Ortuño et al., 2021). Heat treatment is designed to ripen the chicken claws, giving them an attractive color and unique taste, as well as to ensure the hygiene and safety of the product, to achieve preservative effect, and to extend the shelf life. At the same time, it has also been found that inappropriate cooking conditions can destroy some vitamins and minerals and reduce moisture content (Yarmand et al., 2013). For chicken claws, due to its special tissues’ structure and thinner muscle characteristics, the general choice is to cook chicken claws directly in hot water; when the chicken claws are suddenly heated, the surface structure of the skin of the chicken claws gets tighter, which improves the ability to retain water, so that the chicken claw is more tender and juicy (Wang et al., 2019). The cooking method has a great impact on the quality of meat products; however, the heating parameters, such as temperature and time of treatment, also have a considerable impact on the quality of meat (Ceylan & Gokoglu, 2022). In production practice, it is necessary to use appropriate cooking conditions and cooking methods to ensure the quality and nutritional value of chicken claw products.

Myofibrillar proteins (MPs) are the main functional components of meat, which are responsible for the functional properties of processed meat products (Zheng et al., 2019). They are mainly composed of myosin, actin, and actinoglobulin, and their heat‐induced gelation is closely related to both physicochemical and functional properties of meat products (Chen et al., 2018). Myofibrillar protein has a good gelling potential, and during the heating process, the protein denatures, unfolds, and aggregates to form a new stable polymer protein system, and produces a good texture (Xia et al., 2018). Wei et al. (2022) investigated the mechanism of gel formation of chicken MPs under different microwave heating times, and found that prolonging the heating time resulted in the degradation in the quality of the MP gel. Zhang et al. (2024) found that increased temperature in thermal processing may accelerate the oxidation of myofibrillar proteins, with an increase in carbonyl content and a decrease in the total sulfhydryl content.

The aim of this paper is to investigate the effects of cooking conditions with different time–temperature combinations on the physicochemical properties, texture attributes, lipid oxidation, and protein oxidation of chicken claws. This study can reveal the quality loss of chicken claws during thermal processing and provide a theoretical basis for the rational processing of chicken claw products.

## 2 MATERIALS AND METHODS

### 2.1 Materials

In this study, frozen chicken claws (23 ± 3 g) from yellow‐feathered broilers were obtained from a local supermarket (Yibin, China) and stored at −18°C for preservation for further use. Furthermore, thiobarbituric acid and urea were purchased from Shanghai Macklin Biochemical Co., Ltd. (Shanghai, China). The other analytical‐grade chemical reagents used were purchased from Chengdu Kelong Chemical Co., Ltd. (Chengdu, China).

### 2.2 Sample preparation

Four hundred fifty thawed chicken claws were cooked in a water bath at 82, 86, 90, 94, and 98°C (chicken claw/water ratio of 1:5, w/v), 18 per group, and each cooking group was cooked for 11, 13, 15, 17, and 19 min. Immediately after the cooking was completed, the chicken claws were put into an ice‐water bath (1:1, w/v) to be cooled down for 10 min. Finally, the chicken claws were taken out, and the sinews and bones were removed, and set aside.

### 2.3 Cooking loss analysis

In this study, the cooking loss was evaluated according to a previous methodology (Abdel‐Naeem et al., 2021). After thawing was completed, the surface of chicken claws was dried and weighed and recorded as *M*_1_. Then the samples were placed in different cooking environments and cooked. After completion of cooking, the samples were removed and cooled to room temperature. They were then dried, weighed, and recorded as *M*_2_, and finally, the rate of loss by cooking was calculated using Equation (1).

(1) $$\text{Cooking loss}\;\left(\%\right)=\frac{M_{1}-M_{2}}{M_{2}}\times 100$$

### 2.4 Color analysis

**FIGURE 1:**
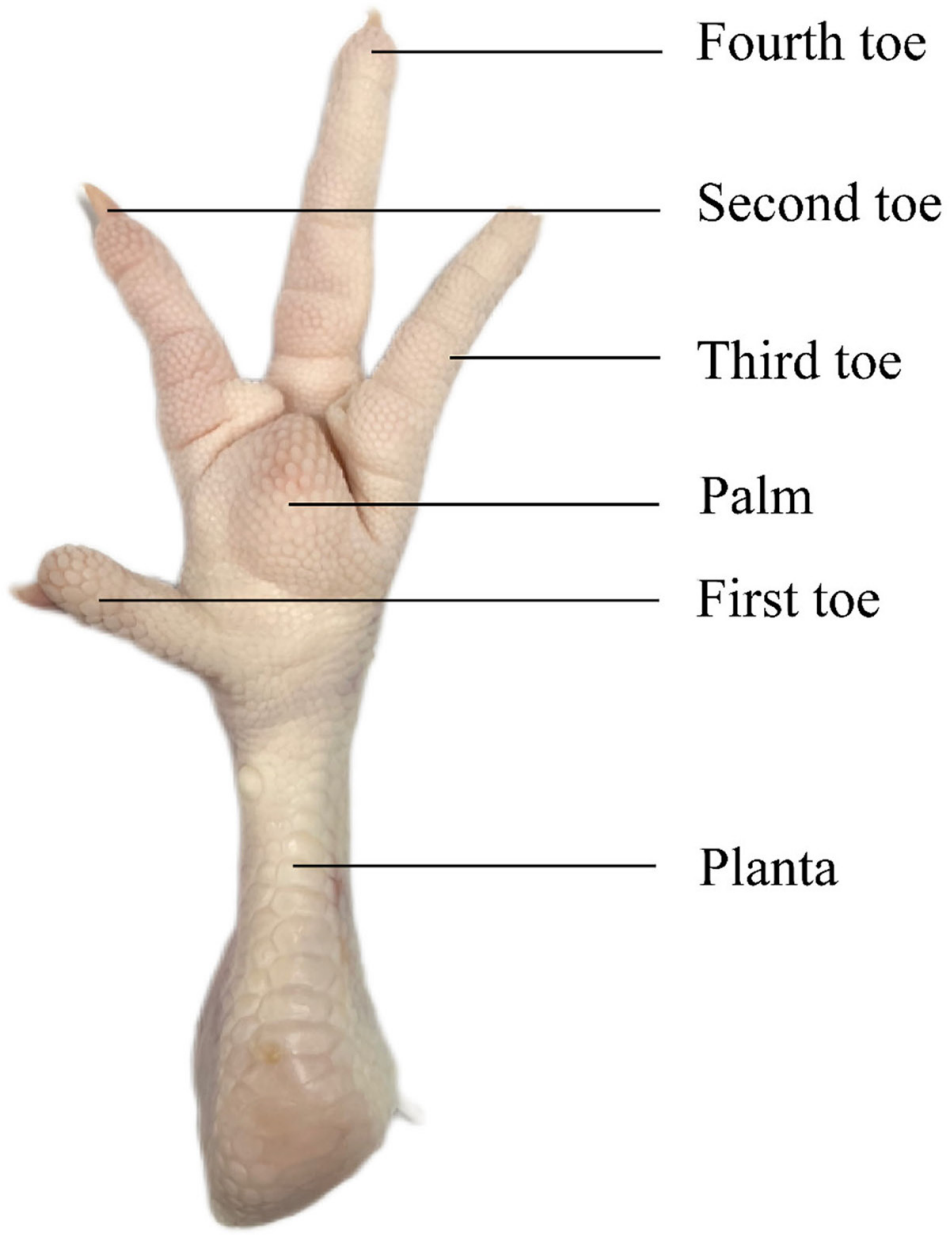
Overview of chicken claws used in this study.

For instrumental color evaluation, a colorimeter (UltraScan VIS, HunterLab, USA) was used to determine the effect of cooking conditions on sample appearance. The planta part of the chicken claw was first selected (Figure 1), and the same position of the sample was placed in the test port of the colorimeter. Then the colorimeter was corrected with a light trap and a white board, selected with a 1‐inch aperture for testing. The color of chicken claw was expressed by L* (brightness), a* (redness/greenness), b* (yellowness/blueness), and ∆E. ∆E was calculated following Equation (2).

(2) $$\mathrm{\Delta E}={\left[{\left(\Delta L^{*}\right)}^{2}+{\left(\Delta a^{*}\right)}^{2}+{\left(\Delta b^{*}\right)}^{2}\right]}^{\frac{1}{2}}$$

In the formula, $∆L^{*}=L_{1}^{*}-L_{0}^{*}$, $∆a^{*}=a_{1}^{*}-a_{0}^{*}$, and $∆b^{*}=b_{1}^{*}-b_{0}^{*}$. $L_{0}^{*}$, $a_{0}^{*}$, and $b_{0}^{*}$ indicate the color of chicken claws before cooking, $L_{1}^{*}$, $a_{1}^{*}$, and $b_{1}^{*}$ indicate the color of chicken claws after cooking.

### 2.5 Puncture force analysis

In this study, the same location of the planta part of the chicken claws was selected for the puncture test by means of a TA‐XT plus texture analyzer (Stable Micro System Co., Ltd., Leicestershire, UK) equipped with a P/2N puncture probe. The test mode was selected as TPA mode, the target mode was Distance, the Distance was 10 mm, the pre‐test speed was 1 mm/s, the test speed was 1 mm/s, and the post‐test speed was 5 mm/s. In this experiment, the same position in the planta of the chicken claw was selected for the puncture test, which was relatively homogeneous.

### 2.6 Shear force analysis

Shear force determinations were made with reference to previous methods with some modifications (Yuan et al., 2023), and tests were performed at the same location on the fourth toe. A TA‐XT plus texture analyzer (Stable Micro System Co., Ltd., Leicestershire, UK) fitted with an HDP/BSW probe was used for testing. The parameters were: pre‐test speed of 2.00 mm/s, test speed of 2.00 mm/s, post‐test speed of 10 mm/s, and target mode tuned to Distance and Distance of 42.00 mm.

### 2.7 Thiobarbituric acid reactive substances (TBARS) analysis

The TBARS parameters were modified somewhat from previous studies (Wu et al., 2016). Briefly, boiled samples were deboned and minced. Then, 10 g of test portion was added to 50 mL of 7.5% (w/w) trichloroacetic acid (TCA) (containing 0.1% (w/w) ethylenediaminetetraacetic acid (EDTA)), shaken for 30 min, and filtered twice using medium‐speed filter paper. Then 5 mL of the supernatant was mixed with 5 mL of 0.02 mol/L thiobarbituric acid (TBA) and placed in a water bath at 95°C for 40 min. The mixture was placed in an ice bath for 5 min, centrifuged at 1600 ***g*** for 5 min, and shaken with 5 mL of chloroform. The supernatant was collected and the absorbance was measured at 532 and 600 nm (UV‐1900i, Shimadzu Co., Ltd., Kyoto, Japan). In this assay, the amount of substance reacted with TBA was expressed as the mass of malondialdehyde (MDA) per 1 kg of sample. Finally, the TBARS value was calculated according to Equation (3):

(3) $$\text{TBARS}\;\left(\text{mgMDA}/\mathrm{kg}\right)=\frac{A_{532\mathrm{nm}}-A_{600\mathrm{nm}}}{155}\times \frac{1}{10}\times 72.6\times 100$$

where “10” is 10 g of sample; “72.6” is the relative molecular weight of MDA; and “155” is the molar absorption coefficient (L/(mol·cm)).

### 2.8 MFI analysis

Myofibrillar fragmentation index (MFI) was determined, as previously described with some adjustments (Aroeira et al., 2020). For this purpose, chicken claws were stripped of bone and tendon and 4 g was used. After that, 15 mL of pre‐cooled MFI buffer (containing 100 mmol/L potassium chloride (KCL), 20 mmol/L potassium phosphate (KH_2_PO_4_), 0.1 mmol/L EDTA, and 1 mmol/L calcium chloride solution, pH = 7.0) was added to the samples. The samples were then homogenized three times at 10,000 r/min for 30 s at 1‐min intervals under an ice bath. After homogenization, the buffer solution was added until a volume of 40 mL was obtained, followed by freezing centrifugation (1000 ***g***, 15 min at 4°C). Then, the supernatant was discarded. Later, the precipitate was resuspended in 40 mL of pre‐cooled MFI buffer and the supernatant was discarded by cryo‐centrifugation (1000 ***g***, 15 min at 4°C). Afterwards, 20 mL of pre‐cooled MFI buffer was added to the precipitate and the suspension was filtered using a 200‐mesh filter cloth. The centrifuge tube was rinsed with 10 mL of pre‐cooled MFI buffer, filtered, and the filtrate was placed in a beaker for further use. In this experiment, the mass concentration of proteins in the filtrate was determined using the bisulfite method, and it was adjusted to 0.5 mg/mL with MFI buffer, and the absorbance was measured at 540 nm. Finally, the value was multiplied by 200 and defined as MFI.

### 2.9 Extraction of myofibrillar protein (MP)

Myofibrillar protein (MP) was extracted with reference to the methods of previous studies and with some adjustments (Yuan et al., 2023). Five grams of boneless chopped chicken claws was taken and extracted with extraction buffer (5 × the volume, 0.1 mol/L sodium chloride (NaCl), 2 mmol/L magnesium chloride (MgCl_2_), 1 mmol/L ethylene glycol tetraacetic acid (EGTA), and 10 mmol/L K_2_HPO_4_, pH = 7.0) and homogenized at 10,000 revolutions/min (rpm). Then, the precipitates were washed using extraction buffer at 2000 g for 10 min at 4°C and the process was repeated four times, until the washing solution was clarified. Afterwards, the solution was filtered using a 200‐mesh filter cloth and the precipitated fraction was dissolved in a buffer solution (containing 20 mmol/L phosphate buffer and 0.6 mol/L NaCl, pH = 7.0). The MP concentration was determined using the bisulfonylurea method using bovine serum albumin (BSA) protein as a standard protein. Finally, the extracted MP was stored at 4°C for further use.

### 2.10 Determination of total sulfhydryl content

The extracted MP solution was diluted to 2 mg/mL, and then 1 mL of the solution was taken and mixed with 8 mL of buffer (0.086 mol/L Tris–HCl, 0.09 mol/L glycine, 4 mmol/L EDTA, and 8 mol/L urea, pH 8), and centrifuged at 10,000 ***g*** for 15 min. After that, 4.5 mL of the supernatant solution was added to 0.5 mL of Ellman's reagent (10 mmol/L 5,5′‐dithiobis‐(2‐nitrobenzoic acid) (DTNB)), and the reaction was carried out at 4°C for 25 min, and the absorbance was measured at 412 nm. The molar extinction coefficient was 13,600 mol/L·cm, and the concentration was determined.

### 2.11 Statistical analysis

All experiments were repeated three times and the results were expressed as mean ± standard deviation (SD). Statistical analysis was performed using SPSS 26 (IBM, Armonk, NY, USA), and significance was analyzed by Duncan's test, with *p* < .05 indicating a statistically significant difference.

## 3 RESULTS AND DISCUSSION

### 3.1 Effect of different cooking conditions on cooking loss rate

**FIGURE 2:**
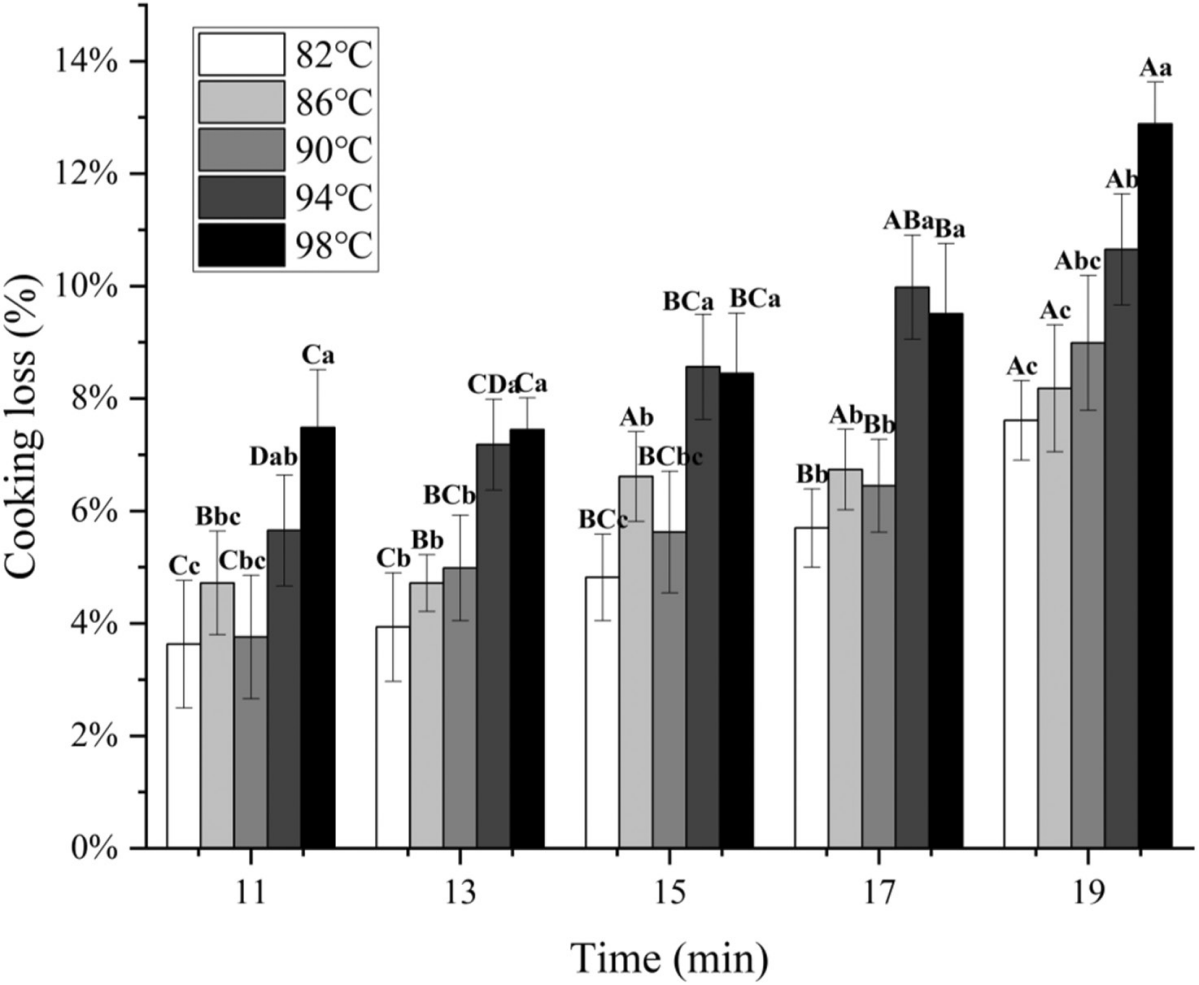
Cooking loss rate of chicken claws under different cooking conditions. Different uppercase letters represent significant differences between different cooking times at the same temperature (*p* < .05); different lowercase letters represent significant differences between different temperatures at the same cooking time (*p* < .05).

In general, the cooking loss rate is closely related to the water retention and juiciness of meat products. Figure 2 shows the results of cooking loss rate of cooked samples. Cooking temperature and time had a significant effect on cooking loss rate (*p* < .05), but the interaction between them was not significant (*p* > .05).

It can be observed that the rate of cooking loss of chicken claws during cooking increases gradually with the increase of temperature and time. At 19 min, the cooking loss rate of chicken claws at 98°C was significantly higher than those at other temperatures (*p* < .05) and increased by 246.46% compared to 11 min at 82°C. This may be due to the boiling water surging under boiling conditions, which generates more bubbles and causes severe physical impact on the chicken claws. At 98°C, the rate of cooking loss of chicken claws cooked for 19 min was significantly higher than those of the remaining four cooking times (*p* < .05), reaching 12.88%. Moreover, there was no significant difference between the 15 min sample and the 11, 13, and 17 min samples (*p* > .05). This indicates that cooking for more than 17 min sample at 98°C can cause serious damage to the texture of chicken claws. During the experiment, it was also observed that after cooking for 19 min at 98°C, the joint area on the back of the chicken claws will break or rupture, which may be due to the expansion of bones and soft tissues inside the claws, resulting in surface rupture. The highest cooking loss rate may be due to the melting and loss of fat at high temperature, as well as the spiral curl transition of collagen during heating, leading to tissue softening, denaturation of actin and myosin, resulting in contraction and fluid loss. In addition, there was no significant difference (*p* > .05) in cooking loss between the samples at 94 and 98°C in the range of 7.18–9.51% at cooking times of 13, 15, and 17 min. In addition, 94 and 98°C were higher than the cooking loss rate of samples at 90°C and below at all cooking times, which may be attributed to the fact that the heating treatment induces an irreversible denaturation of muscle proteins, which is caused by the denaturation of the proteins and an increase in contraction of the myofibrils at the higher temperatures (Nasyiruddin et al., 2021).

### 3.2 Effect of different cooking conditions on color

The color of chicken claws is an important factor in consumer choice. The instrumental color characteristics (L*, a*, b*, ∆*E*) of chicken claws cooked at different combinations of temperature and time are given in Table 1.

**TABLE 1:** Table of determination results of color difference of chicken claws under different cooking conditions.

Temperature	Time	L*	a*	b*	Δ*E*
82°C	0	82.39 ± 0.84^A^	4.25 ± 0.39^A^	9.46 ± 0.70^C^	–
	11	72.53 ± 1.59^Bab^	1.33 ± 0.25^Cb^	15.12 ± 0.99^Aab^	11.79 ± 1.36^Babc^
	13	72.02 ± 1.24^Bab^	0.28 ± 0.07^Eab^	13.04 ± 0.73^ABbc^	11.68 ± 1.29^Bab^
	15	71.27 ± 1.31^Bb^	0.91 ± 0.19^Da^	12.77 ± 0.74^ABbc^	12.11 ± 0.99^Ba^
	17	70.92 ± 0.54^BCab^	1.68 ± 0.06^Ba^	12.35 ± 0.83^ABb^	12.12 ± 0.66^Bab^
	19	68.83 ± 1.12^Cab^	0.33 ± 0.22^Ec^	10.71 ± 3.34^BCab^	14.44 ± 0.94^Aa^
86°C	0	82.39 ± 0.84^A^	4.25 ± 0.39^A^	9.46 ± 0.70^E^	–
	11	72.26 ± 0.76^Bb^	0.11 ± 0.04^Bc^	15.50 ± 1.11^Aa^	12.54 ± 0.04^Ba^
	13	70.86 ± 0.49^Cb^	0.11 ± 0.05^Bb^	12.46 ± 0.72^BCc^	12.62 ± 0.49^Ba^
	15	71.10 ± 0.20^Bbb^	0.22 ± 0.06^Bc^	13.51 ± 0.43^Bab^	12.66 ± 0.08^Ba^
	17	70.54 ± 0.75^Cab^	0.11 ± 0.06^Bd^	12.00 ± 0.50^CDb^	12.81 ± 0.79^Bab^
	19	68.73 ± 1.07^Dab^	−0.33 ± 0.08^Cd^	10.98 ± 0.88^Dab^	14.51 ± 1.01^Aa^
90°C	0	82.39 ± 0.84^A^	4.25 ± 0.39^A^	9.46 ± 0.70^C^	–
	11	74.45 ± 1.00^Ba^	2.25 ± 0.22^Ba^	16.38 ± 0.622^Aa^	10.74 ± 0.95^Bbc^
	13	74.09 ± 1.66^Ba^	0.04 ± 0.03^Eb^	16.21 ± 0.85^Aa^	11.52 ± 1.64^Bab^
	15	72.87 ± 0.33^Bab^	0.59 ± 0.09^Db^	14.76 ± 1.13^Aa^	11.52 ± 0.65^Ba^
	17	71.78 ± 1.31^Cab^	0.68 ± 0.10^Dc^	12.51 ± 1.11^Bb^	11.63 ± 1.45^Bab^
	19	68.16 ± 1.30^Dab^	1.56 ± 0.2^Ca^	12.48 ± 0.94^Bab^	14.83 ± 1.06^Aa^
94°C	0	82.39 ± 0.84^A^	4.25 ± 0.39^A^	9.46 ± 0.70^C^	–
	11	74.49 ± 1.03^Ba^	0.36 ± 0.07^Dc^	15.21 ± 0.28^Aa^	10.53 ± 0.75^Cc^
	13	74.07 ± 1.37^Ba^	0.62 ± 0.15^Ca^	14.38 ± 0.57^ABb^	10.37 ± 0.89^Cb^
	15	71.33 ± 0.92^Cab^	0.60 ± 0.11^Cb^	13.76 ± 0.78^Bab^	12.44 ± 0.58^Ba^
	17	72.60 ± 0.77^Bab^	0.87 ± 0.02^Bb^	14.40 ± 0.39^ABa^	11.48 ± 0.67^BCb^
	19	69.42 ± 1.02^Da^	0.67 ± 0.14^Cb^	13.41 ± 0.67^Ba^	14.03 ± 1.05^Aa^
98°C	0	82.39 ± 0.84^A^	4.25 ± 0.39^A^	9.46 ± 0.70^B^	–
	11	71.66 ± 0.70^Bb^	−0.2 ± 0.82^Bc^	13.50 ± 1.15^Ab^	12.34 ± 0.74^Bab^
	13	70.94 ± 0.73^Bb^	−0.73 ± 0.39^Bc^	12.15 ± 1.17^Ac^	12.81 ± 0.72^Ba^
	15	71.52 ± 0.79^Bab^	−0.37 ± 0.08^Bd^	12.42 ± 1.78^Ab^	12.27 ± 0.55^Ba^
	17	69.91 ± 1.60^Bb^	−0.31 ± 0.10^Be^	11.91 ± 1.48^Ab^	13.58 ± 1.37^Ba^
	19	67.36 ± 0.33^Cb^	−0.48 ± 0.18^Bd^	9.60 ± 0.41^Bb^	15.76 ± 0.37^Aa^

*Note*: Different uppercase letters represent significant differences between different cooking times at the same temperature (*p* < .05); different lowercase letters represent significant differences between different temperatures at the same cooking time (*p* < .05).

As for brightness, cooking time and temperature had a significant effect on chicken claws (*p* < .05), but the interaction between the two was not significant (*p* > .05). According to Table 1, compared with uncooked chicken claws, the L* of cooked chicken claws significantly decreases, ranging from 67.36 to 74.49. Rees et al. (2003) have found that uncooked samples show higher L* because there is more water impregnated on the sample surface. Similarly, it can also be explained that as the cooking time increases, the cooking loss rate increases, while L* gradually decreases. This is because the decrease in sample moisture leads to a lower surface glossiness of the samples.

In addition, cooking time and temperature had a significant effect on the yellowness of chicken claws (*p* < .05), but their interaction was not significant (*p* > .05). As the cooking time increases, the b* value shows a gradually decreasing trend. In this case, it is noteworthy that a significant increase in b* was observed in chicken claws after 11 min of cooking compared to uncooked samples (*p* < .05). The explanation given in the previous study was that the increase in b* value might be related to the increase in lipid oxidation, and this explanation is also in agreement with the results of the TBARS values in this paper (Wang et al., 2018). However, with the increase in cooking time, the b* was decreased, which might be due to the fact that the chicken claws themselves contain less fat, and the increase in cooking time further leads to the degradation and leaching of some proteins, which in turn leads to a decrease in yellowness. This is in contrast to previous findings on other meat products, where Becker et al. (2015) found that the brightness value and yellowness of pork M. longissimus thoracis et lumborum increased with increased heating time.

In terms of redness, cooking time and temperature as well as their interaction had a significant effect (*p* < .05) on chicken claw samples. It can be observed from Table 1 that compared with the uncooked samples, the a* values underwent a significant decrease; this is because, myoglobin denatures between 55 and 65°C, making the samples less reddened, but unlike other meat products. Yuan et al. (2023) concluded that the chicken claw samples had less myoplasmic proteins, and more myofibrillar proteins and matrix proteins, while myoglobin and other substances contained in myoplasmic proteins are closely related to the redness value. Therefore, after cooking for more than 11 min, there was no significant change in a*. In addition, the a* of the chicken claw samples under 98°C boiling condition all showed negative values, which imply that the high temperature boiling condition caused the chicken claws to lose their sense of redness.

The results of the total color difference between raw and cooked chicken claws are shown in Table 1. Cooking time and temperature and their interaction had a significant effect on the chicken claw samples (*p* < .05). When ∆*E* >5, the color difference between meat products is noticeable and the observer can notice two different colors (Ruedt et al., 2023); as can be seen in Table 1, the total color difference is between 10.37 and 15.76, and therefore perceptible to humans. The total color difference of the chicken claw samples cooked for 19 min at the same temperature reached the maximum.

### 3.3 Puncture force analysis under different cooking conditions

**FIGURE 3:**
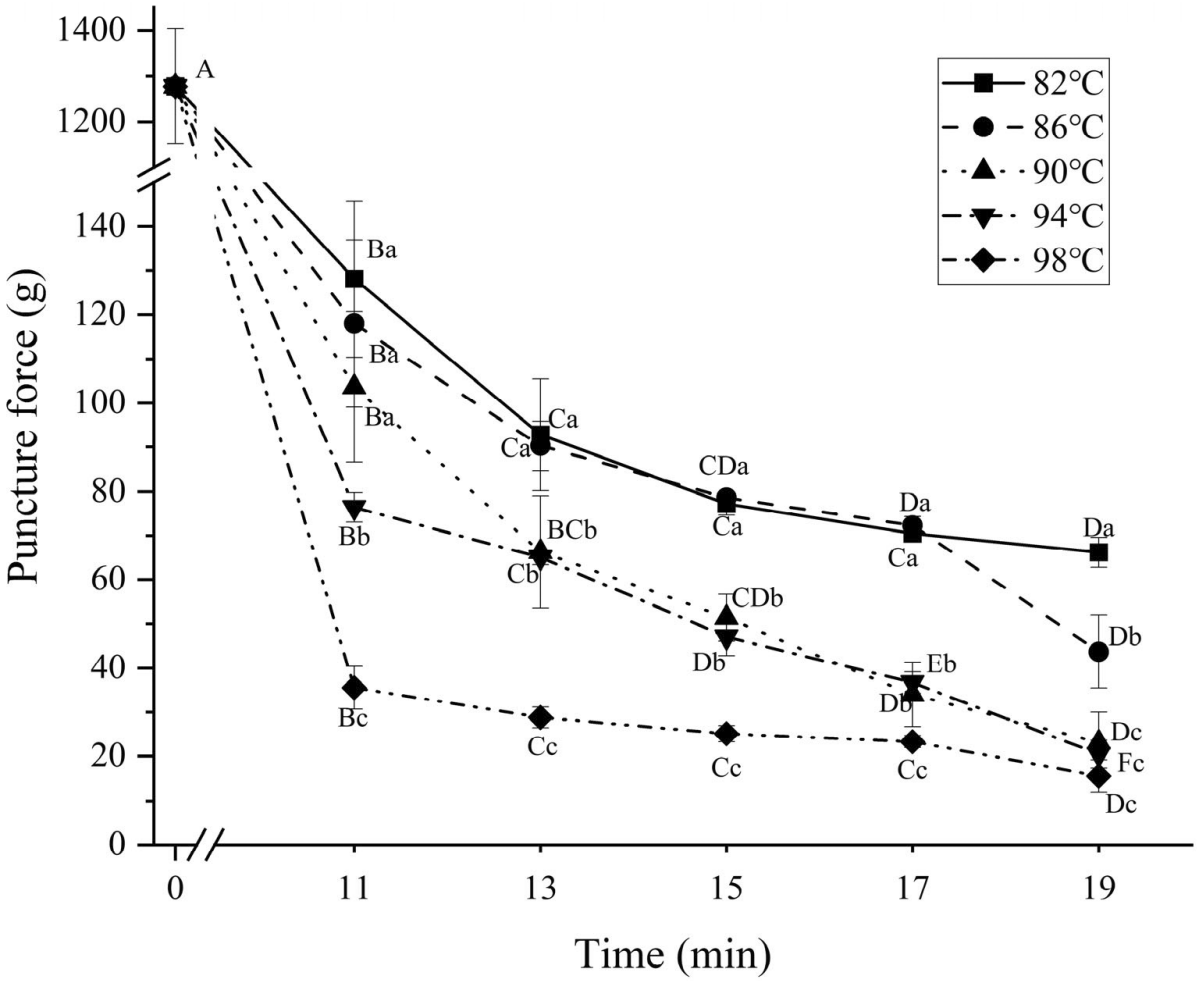
Puncture forces of chicken claws under different cooking conditions. Different uppercase letters represent significant differences between different cooking times at the same temperature (*p* < .05); different lowercase letters represent significant differences between different temperatures at the same cooking time (*p* < .05).

The puncture force obtained by the puncture method of texture analyzer can reflect well the gel strength and the tenderness of meat products. Moreover, the shape and size of the chicken claw samples differed from one part to another, so more accurate tenderness data could be obtained through the penetration experiment (Luyten et al., 1992). The puncture force results of chicken claws cooked at different temperature and time combinations are shown in Figure 3. The effects of cooking temperature and time on puncture force are significant (*p* < .05), and there is a significant interaction (*p* < .05).

As can be seen from Figure 3, on the whole, the puncture force of the cooked samples was in the range of 15.55–128.10 g. And the puncture force of the samples gradually decreased with the increase of cooking temperature and time, attributed to the increase of the thinning degree of myogenic fibers and the precipitation of collagen (Tang et al., 2023). Compared with the uncooked sample (with a puncture force of 1277.47 g), the puncture force of the cooked samples significantly decreased by more than 1000 g (*p* < .05). This is because cooking causes cell rupture in the chicken claws, protein denaturation in the intercellular matrix, molecular chains becoming loose, and the cooking process reduces the degree of collagen cross‐linking in the chicken claws. Some soluble collagen gradually dissolves, resulting in a decrease in the strength and toughness of the chicken claws, making it easier for the probe to penetrate the internal matrix structure of the chicken claws (Tang et al., 2023). The puncture force of all samples cooked at 98°C was below 40 g, which was significantly lower than that of the samples cooked at other temperatures under the same cooking time (*p* < .05), and the overall change of the penetration force with the increase of time was relatively smooth. This may be due to the fact that the spatial conformation of the proteins has been severely damaged during cooking at 98°C, and the chemical bonds and groups such as hydrogen and disulfide bonds that maintain the spatial structure have been oxidized and destroyed (Wang et al., 2023).

### 3.4 Shear force analysis under different cooking conditions

**FIGURE 4:**
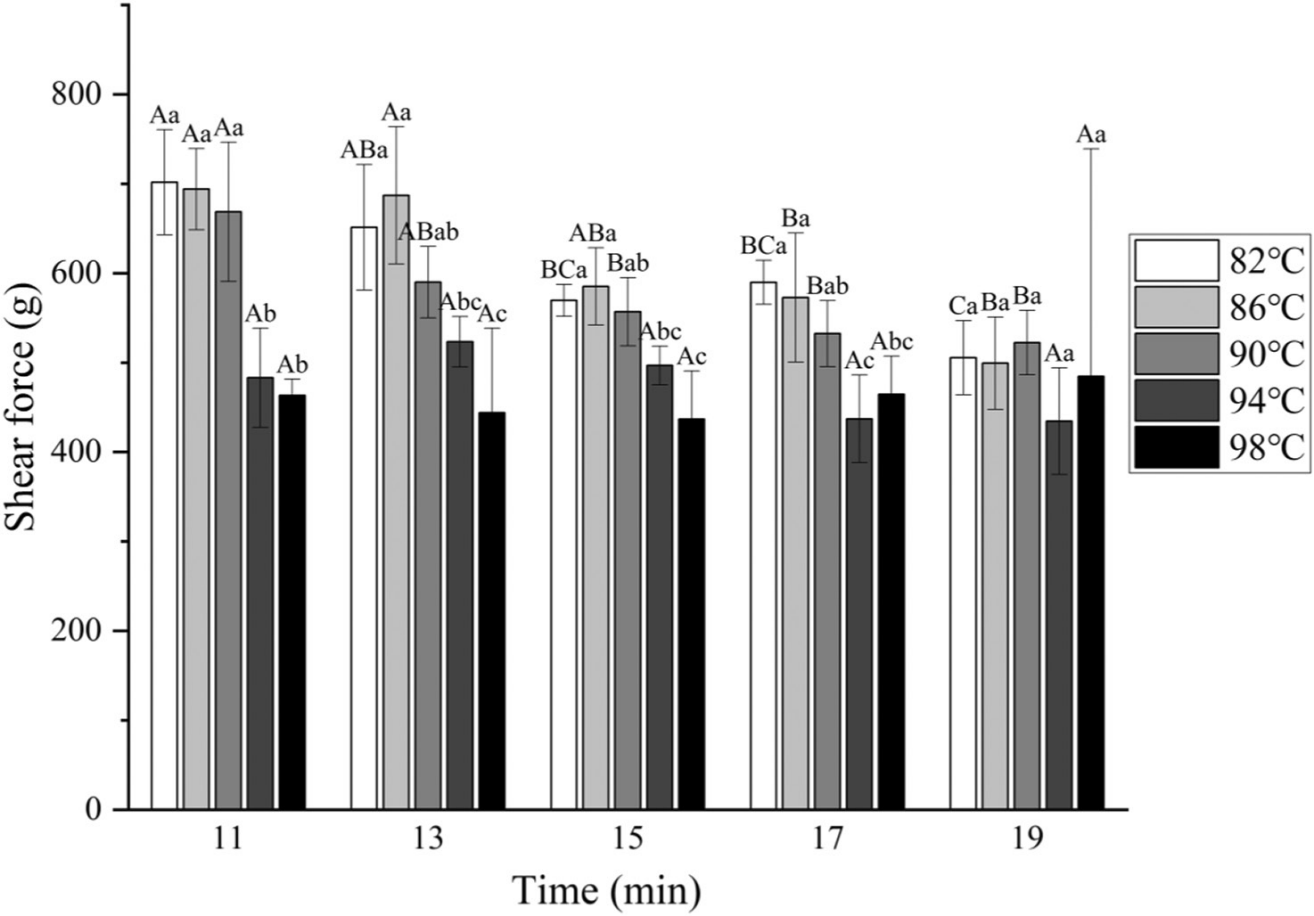
Shear forces of different parts of chicken claws under different cooking conditions. Different uppercase letters represent significant differences between different cooking times at the same temperature (*p <* .05); different lowercase letters represent significant differences between different temperatures at the same cooking time (*p* < .05).

In the study of meat texture, shear force is usually considered to be the main determinant of meat tenderness, and the shear force used in cutting meat products is generally regarded as the tenderness of the meat, and the higher the shear force used, the less tender the meat will be (Contreras‐Lopez et al., 2020). The results of shear force of chicken claw samples cooked at different combinations of time and temperature are shown in Figure 4. Both cooking time and temperature had a significant effect on the results (*p* < .05), but the interaction between the two was not significant (*p* > .05). The shear force value of uncooked chicken claw samples was 8925.8 g. The shear force exhibited was higher due to the connective tissue consisting of more complete collagen fibers with higher degree of densification. Whereas after cooking the sample shear force showed a significant decrease (*p* < .05), Chakka et al. (2017) also reported that the gel strength of chicken claw collagen starts to decrease when the water bath temperature reaches above 60°C. This is because too much heat causes the hydrogen bonds between the protein molecules to break, and the tertiary and secondary structures of proteins to be destroyed, and at the same time, the gel starts to melt, and many other factors lead to the meat product shear force decreases. As a whole, the shear force of chicken claw samples showed a gradual decrease with increasing cooking temperature and time. The structure and composition of chicken claw itself not only gets damaged when special, water bath heating process is employed to make its collagen dissolve, the myofibrillar protein's crosslinking structure is also destroyed, and then the overall toughness and strength of the chicken claw declined, with the shear force also decreasing. This is slightly different from other meat products. Wang et al. (2023) found that at the initial stage of cooking of braised meat, the shear force would keep on rising, and they attributed this phenomenon to the denaturation of MPs, which led to the contraction of muscle fibers and the decrease of water‐holding capacity. After 20 min of cooking, the shear force values showed a decreasing trend, which was explained by the dissolution of connective tissue and structural damage of MPs. It can be observed that when cooked below 90°C, the contraction process of muscle proteins inhibits the decrease of shear force to a certain extent, so in the table it will be found that the level of shear force of samples cooked at 86°C is comparable to the level of shear force of samples cooked at 82°C for the same time. Previous studies have reported the effect of cooking temperature on MPs, and the optimal three‐dimensional (3D) network structure and gel properties of MP can be formed at higher than 70°C (Du et al., 2021).

### 3.5 Effect of different cooking conditions on lipid oxidation

**FIGURE 5:**
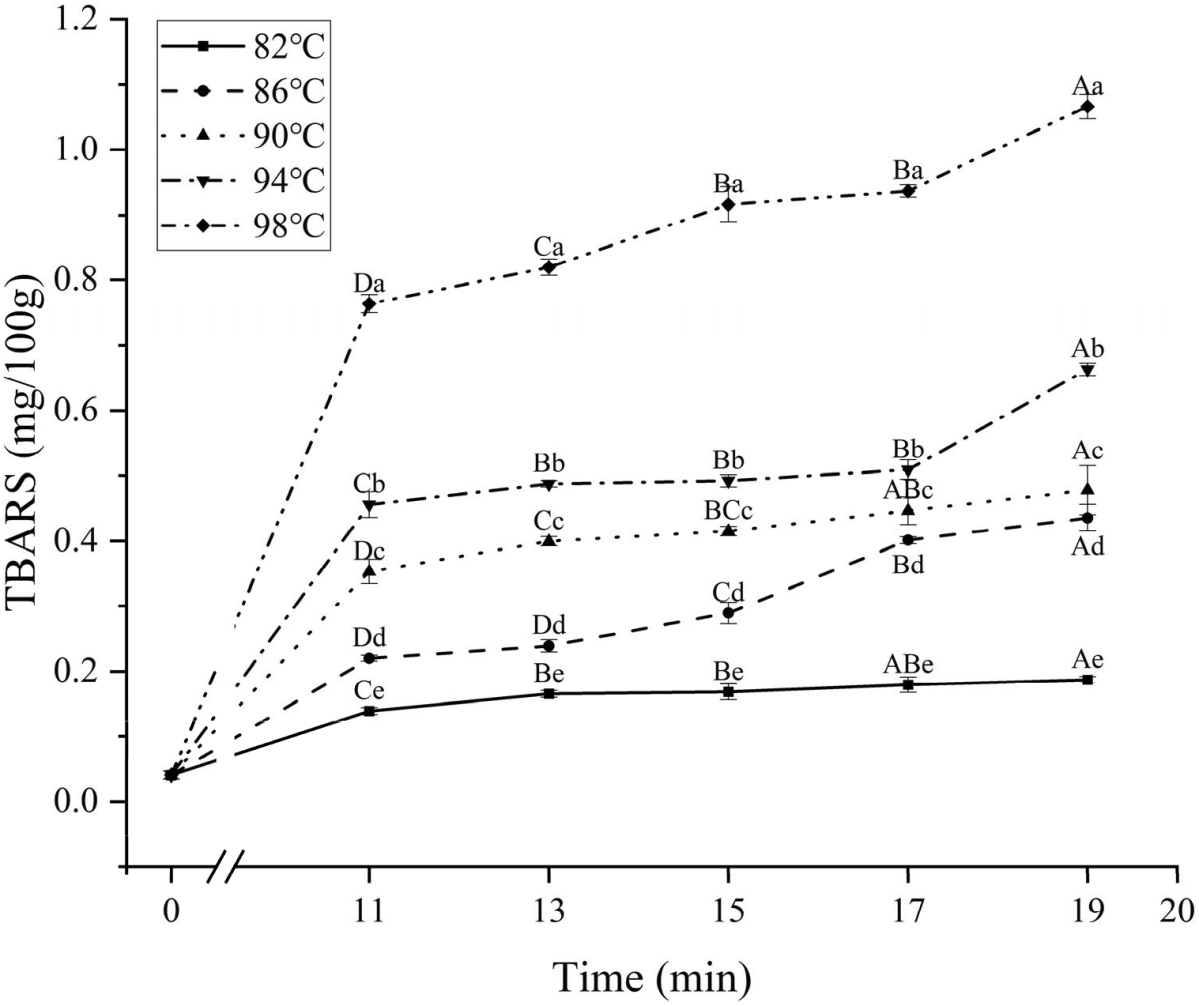
The TBARS values of chicken claws under different cooking conditions. Different uppercase letters represent significant differences between different cooking times at the same temperature (*p* < .05); different lowercase letters represent significant differences between different temperatures at the same cooking time (*p* < .05).

The results of TBARS values of chicken claws treated by cooking at different combinations of temperature and time are shown in Figure 5. Both cooking time and temperature as well as the interaction between the two had a significant effect on TBARS (*p* < .05).

Thiobarbituric acid reactive substances (TBARS) are one of the typical indicators for assessing the content of secondary oxidation products of lipids (e.g., aldehydes) in meat products (Bindu et al., 2013). In previous studies, it has been found that water bath cooking is the least fat oxidized of the five basic cooking methods (Abdel‐Naeem et al., 2021). According to Figure 5, it can be seen that the TBARS values of the boiled samples all showed a significant increase compared to the unboiled samples, and a highly significant increase (*p* < .01) was observed at 98°C, reaching 0.76 mg/100 g for the sample boiled for 11 min. This may be due to the fact that, in the boiled state, the lipids react vigorously and the rate of lipid oxidation increases at the same time, resulting in rapid decomposition of the primary products from oxidation into secondary products such as malondialdehyde. In addition, significant differences (*p* < .05) were observed in the samples at each temperature within five identical cooking times, indicating that temperature has a strong influence on the degree of lipid oxidation in chicken claw samples. It is noteworthy that the significant increase in TBARS value of the 82°C samples began to pause after cooking for more than 13 min (*p* > .05), and, the TBARS value was only 0.19 mg/100 g when it was cooked for 19 min, which was not yet up to the value of 0.22 mg/100 g when it was cooked at 86°C for 11 min.

On the whole, compared with other meat products, the degree of lipid oxidation of chicken claws after cooking was relatively low, first, because the lipid content of chicken claws was less, and the reactants involved in lipid oxidation were correspondingly less, and the appropriate heating method, heating temperature and time would passivate the enzymes in the meat products and slow down the oxidative decay of the meat itself; and second, because the cooking time of this experiment was shorter as a whole, and the indexes were started immediately after the cooking measurement, which did not leave the chicken claws in an environment and state of continuous high‐intensity oxidation.

### 3.6 Effect of different cooking conditions on MFI

**FIGURE 6:**
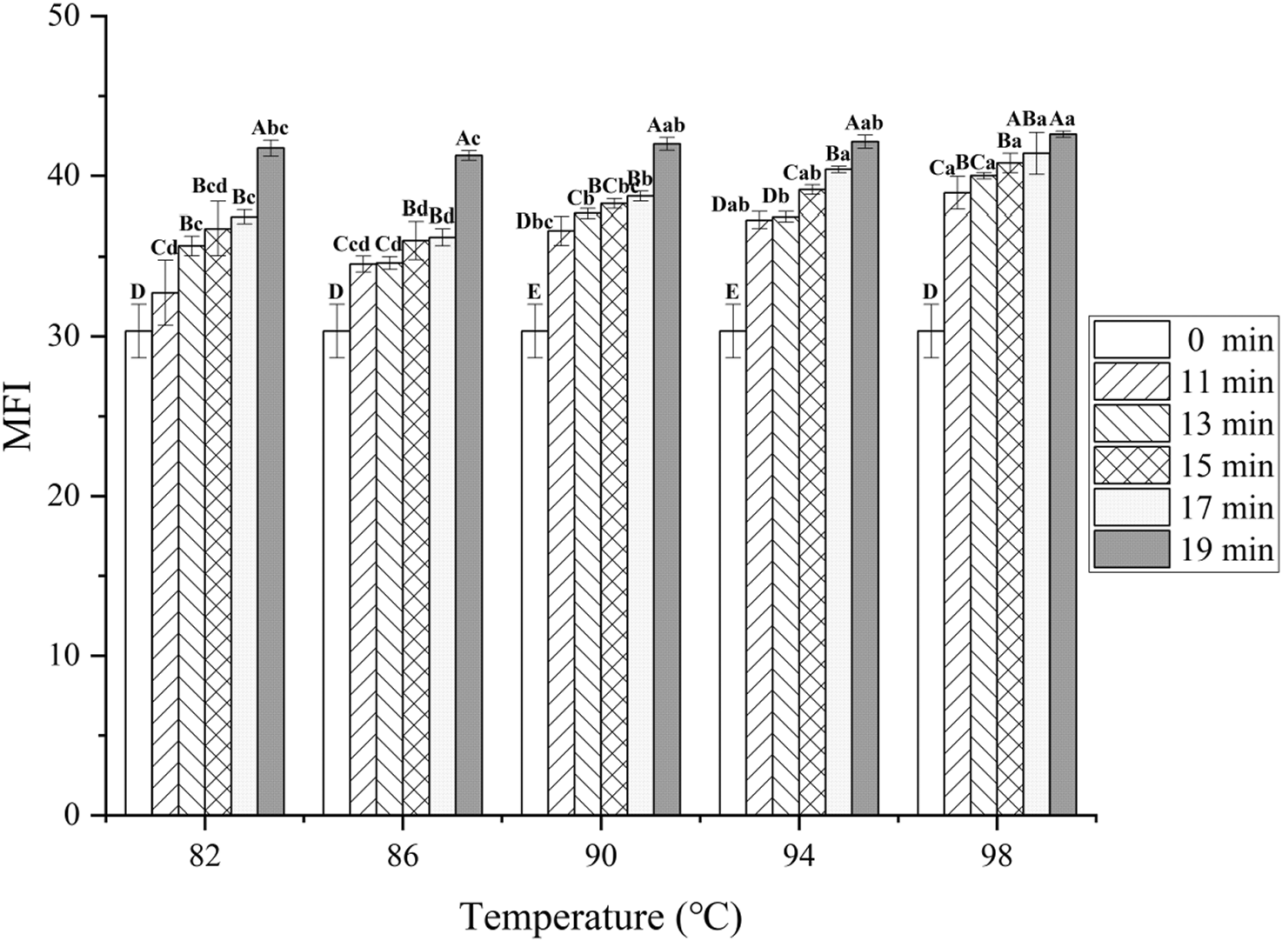
Effects of different cooking conditions on MFI of chicken claws. Different uppercase letters represent significant differences between different cooking times at the same temperature (*p* < .05); different lowercase letters represent significant differences between different temperatures at the same cooking time (*p* < .05).

Figure 6 shows the results of MFI values of chicken claws cooked at different combinations of time and temperature. Both studied factors were significant (*p* < .05) with significant interaction (*p* < .05) on MFI values. The MFI reflects the degree of degradation of MPs and their structural destruction, and the larger the MFI value, the better the muscle tenderness. Usually, the higher the MFI value, the higher the degree of fragmentation of myogenic fibers, so it can be assumed that the MFI value is positively correlated with the tenderness of meat (Li et al., 2018). As can be seen from Figure 6, compared with the uncooked samples, the samples after 11 min of cooking all showed significant increases (*p* < .05), and the MFI showed an overall increasing trend with the increase of cooking time and temperature, indicating that water bath cooking has a certain degree of tenderizing effect on chicken claws. Currently, there are fewer studies on the effect of cooking time and temperature on myogenic fiber shrinking of meat products, and more on the effect of post‐slaughter aging process on meat products, but its MFI was about 30 at day 0 post‐slaughter, which is consistent with the results of this study (Luo et al., 2022). Except for the 98°C condition, significant elevation (*p* < .05) occurred in the samples cooked for 19 min compared to those cooked for 17 min, suggesting that cooking for 19 min causes further rupture and dissolution of myogenic fibers in chicken claws. On the other hand, at the same cooking time, the MFI of the 98°C cooking group was the highest compared with all other temperatures, which indicated that the elevated temperature could deepen the degree of destruction of myofibril structure.

### 3.7 Effect of different cooking conditions on the total sulfhydryl content in MPs

**FIGURE 7:**
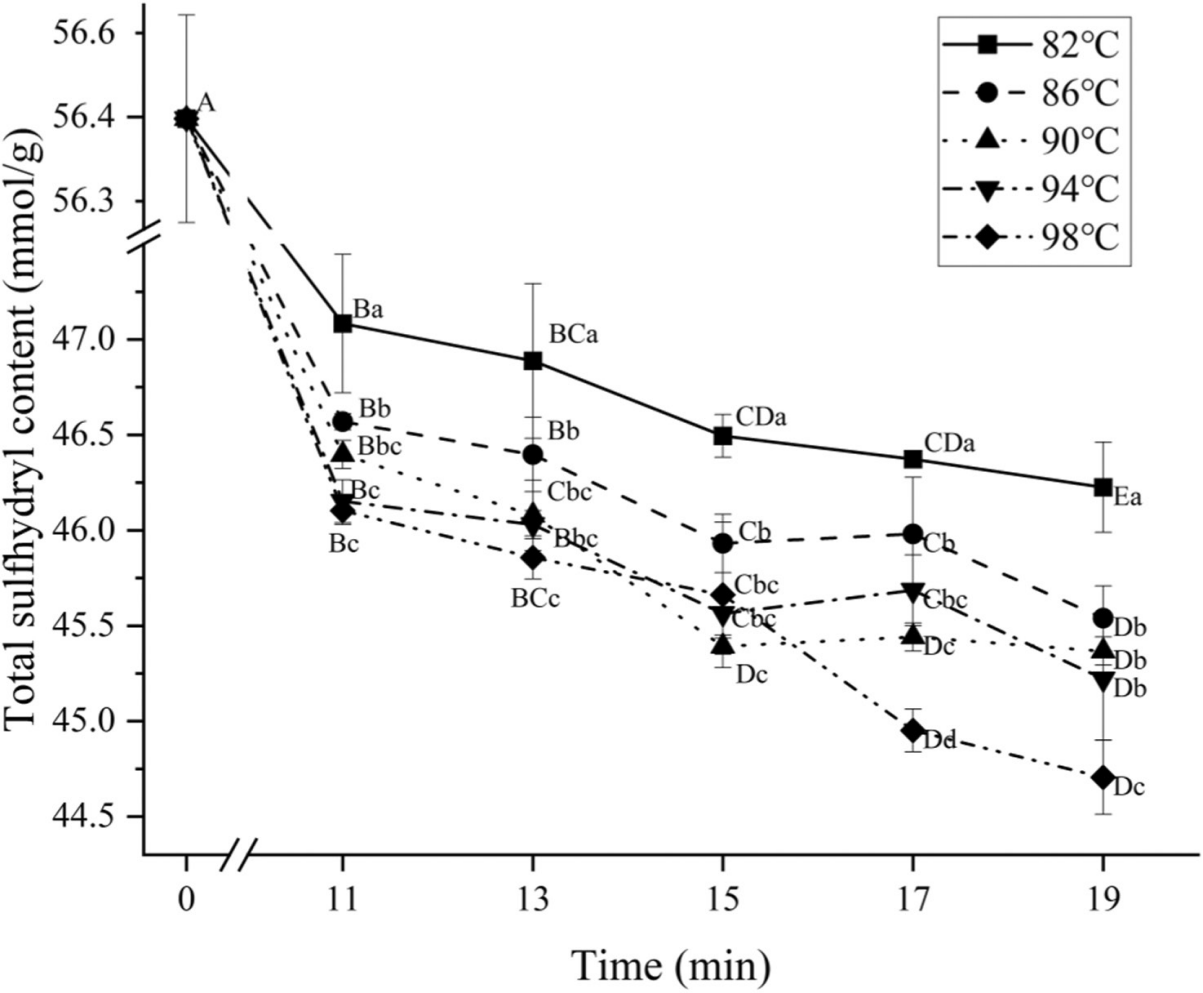
Total sulfhydryl content of chicken claws' myofibrillar protein under different cooking conditions. Different uppercase letters represent significant differences between different cooking times at the same temperature (*p* < .05); different lowercase letters represent significant differences between different temperatures at the same cooking time (*p* < .05).

The results of total sulfhydryl content of chicken claws cooked at different combinations of temperature and time are shown in Figure 7. Cooking time and temperature had a significant effect (*p* < .05) on total sulfhydryl content with a significant interaction (*p* < .05). MPs are rich in sulfhydryl groups, which play an important role in measuring the degree of oxidation and maintaining protein conformation during heat treatment. During the heating process, when the two cysteine molecules on the adjacent protein chain are oxidized, the sulfhydryl group (‐SH) will disappear and some will form a disulfide bond (S‐S) between the molecules. The decrease of ‐SH group itself will change the conformation of protein, and the formation of disulfide bond will further lead to the formation of protein aggregation structure (Chen et al., 2019). As can be seen in Figure 7, the total sulfhydryl content of chicken claw samples gradually decreased with increasing time and temperature throughout the cooking, which indicates that the protein conformation has been changing during the heating treatment. The overall change in range was only 11.70 mmol/g MP, which was smaller than the decrease of 20 nmol/mg between raw meat and 80°C heating temperature found by (Yu et al., 2021). Compared with the uncooked samples, the total sulfhydryl content of the samples after 11 min of cooking all underwent a significant decrease (*p* < .05), but there was no significant difference between the 90°C cooking group and 86, 94, and 98°C (*p* > .05), which may be attributed to the fact that some sulfhydryl groups in the MPs were facilitated to disappear and disulfide bonds were generated in this heating treatment, which caused the protein to gradually undergo aggregation slowing down the reaction of some of the internal sulfhydryl group reactions (Zhu et al., 2019). The same phenomenon happened not only at 13 min, but also at 15 min, the total sulfhydryl group of the 90°C sample was the lowest among all temperatures, which was only 45.39 mmol/g MP. There was no significant difference between 86, 90, and 94°C at 19 min (*p* > .05), but a significant decrease to 44.71 mmol/g MP occurred at 98°C (*p* < .05).

It is noteworthy that the total sulfhydryl groups of the samples tended to increase slightly compared to the total sulfhydryl groups of the samples between 15 and 17 min when cooked at 86, 90, and 94°C temperatures. This development was possibly due to the fact that the disulfide bonds, which had formed a stable structure on the surface of the chicken claw MPs prior to the 15 min of cooking, were disrupted and the sulfhydryl groups were re‐formed between 15 and 17 min (Ishiwatari et al., 2013). In contrast, the total sulfhydryl groups of the samples cooked at 98°C for 17 min decreased significantly to 44.95 mmol/g MP, which suggests that the heat treatment effect is too strong, causing the sulfhydryl groups to be continuously destroyed by oxidation.

## 4 CONCLUSION

Cooking treatment had a significant effect on the quality characteristics of chicken claws, and the cooking time and temperature had a significant effect on the rate of cooking loss, color, textural characteristics, degree of lipid oxidation, MFI, and total sulfhydryl content of chicken claws. The results showed that the cooking loss rate, degree of lipid oxidation, and MFI value of chicken claws gradually increased, and the total color difference, puncture force, shear force, and total sulfhydryl content gradually decreased with the increase of cooking temperature and cooking time. Although chicken claws cooked at higher times and temperatures had better texture, the loss of juiciness, color, and nutrients meant that these chicken claws did not necessarily satisfy consumer demand, and a compromise between these parameters had to be found in order to obtain a high‐quality chicken claw product. Overall, chicken claws cooked at 86, 90, and 94°C for 15–17 min had better texture and flavor. Further work needs to be carried out with a view to perfecting the determination of the optimum cooking conditions for chicken claws and providing a scientific theoretical basis for the further processing of chicken claw products.

## AUTHOR CONTRIBUTIONS

**Yifan Yu:** Conceptualization (equal); data curation (equal); formal analysis (equal); funding acquisition (equal); investigation (equal); methodology (equal); project administration (equal); resources (equal); software (equal); supervision (equal); validation (equal); visualization (equal); writing – original draft (equal); writing – review and editing (equal). **Xianling Yuan:** Conceptualization (equal); data curation (lead); formal analysis (lead); funding acquisition (lead); investigation (lead); methodology (supporting); project administration (lead); resources (lead); software (supporting); supervision (supporting); validation (supporting); visualization (supporting); writing – original draft (supporting); writing – review and editing (lead). **Zhouyou Zhang:** Resources (supporting); software (supporting); supervision (supporting); validation (equal); writing – original draft (equal). **Yidan Zheng:** Data curation (equal); investigation (equal); methodology (equal); software (equal); validation (equal). **Ying He:** Conceptualization (equal); data curation (equal); formal analysis (equal); resources (equal); validation (equal). **Yingru Zhou:** Conceptualization (equal); data curation (equal); formal analysis (equal); resources (equal); validation (equal).

## FUNDING INFORMATIONS

This work received funds from the Science and Technology Department of Sichuan Province [2020YFN0151].

## CONFLICT OF INTEREST STATEMENT

The authors declare that they do not have any conflict of interest.

## ETHICS STATEMENT

This study does not involve any human or animal testing.

## INFORMED CONSENT

Written informed consent was obtained from all study participants.

## Data Availability

Research data are not shared.
